# Crystal Structure of the Isocitrate Dehydrogenase 2 from *Acinetobacter baumannii* (AbIDH2) Reveals a Novel Dimeric Structure with Two Monomeric-IDH-Like Subunits

**DOI:** 10.3390/ijms19041131

**Published:** 2018-04-10

**Authors:** Peng Wang, Yatao Wu, Jie Liu, Ping Song, Shan Li, Xinxin Zhou, Guoping Zhu

**Affiliations:** 1 Anhui Provincial Key Laboratory of the Conservation and Exploitation of Biological Resources, College of Life Sciences, Anhui Normal University, Wuhu 241000, China; wangpeng@ahnu.edu.cn (P.W.); wuyuntian36@hotmail.com (Y.W.); liujie2017@ahnu.edu.cn (J.L.); Shanli2016@yahoo.com (S.L.); 18817376223@163.com (X.Z.); 2 College of Biological and Chemical Engineering, Anhui Polytechnic University, Wuhu 241000, China; songping1987@foxmail.com

**Keywords:** isocitrate dehydrogenase, crystal structure, *Acinetobacter baumannii*, dimerization, thermostability, phylogenetic studies

## Abstract

Monomeric isocitrate dehydrogenases (IDHs) have a single polypeptide sizing around 85 kDa. The IDH2 from the opportunistic bacterium *Acinetobacter baumannii* (AbIDH2) with a mass of 83 kDa was formerly recognized as a typical monomeric IDH. However, both size exclusion chromatography and analytical ultracentrifugation analysis indicated that AbIDH2 exists as a homodimer in solution. The crystallographic study of the substrate/coenzyme-free AbIDH2 gave a dimeric structure and each subunit contained a domain I and a domain II. The dimeric assembly is mainly stabilized by hydrophobic interactions (16 hydrogen bonds and 11 salt bridges) from the dimer’s interface platform, which centered around the three parallel helices (α4, α12, and α17) and one loop from the domain II. Kinetic analysis showed that the dimeric AbIDH2 showed much lower catalytic efficiency (0.39 μM^−1^·s^−1^) as compared to the typical monomeric IDHs (~15 μM^−1^·s^−1^). Key residues crucial for dimer formation were simultaneously changed to generate the mutant mAbIDH2. The disruption of the hydrophobic forces disassociated the dimeric AbIDH2, making mAbIDH2 a monomeric enzyme. mAbIDH2 sustained specific activity (21.9 ± 2 U/mg) comparable to AbIDH2 (25.4 ± 0.7 U/mg). However, mAbIDH2 proved to be a thermolabile enzyme, indicating that the thermostable dimeric AbIDH2 may have a physiological significance for the growth and pathogenesis of *A*. *baumannii*. Phylogenetic analysis demonstrated the existence of numerous AbIDH2 homologous proteins, thus expanding the monomeric IDH protein family.

## 1. Introduction

Isocitrate dehydrogenase (IDH) catalyzes the NAD(P)^+^-dependent oxidative decarboxylation of isocitrate to α-ketoglutarate (α-KG) and the NAD(P)H/CO_2_-dependent reductive carboxylation of α-KG to isocitrate. Two types of IDHs exist in nature according to the coenzyme specificity: NAD^+^-dependent IDHs (EC 1.1.1.41, NAD-IDHs) and NADP^+^-dependent IDHs (EC 1.1.1.41, NADP-IDHs). NADH generated by NAD-IDHs is the main component of energy metabolism, while NADPH generated by NADP-IDHs is an important source of reducing power. NADPH also serves as a defense against oxidative damage in vivo [1,2,3].

Four kinds of IDHs with different oligomeric states have been found in nature: monomeric IDHs, homo-dimeric IDHs, homo-tetrameric IDHs, and hetero-oligomeric IDHs [4,5,6]. When taking into consideration the coenzyme specificity, the IDH protein family can be very divergent. Our previous works have redefined the phylogenesis of the IDH protein family [7,8,9]. Three diverse phylogenetic subfamilies can be distinguished among all IDHs. Type I IDHs are composed of homo-dimeric NAD(P)-IDHs from archaea and bacteria, as well as homo-tetrameric NAD(P)-IDHs from bacteria and hetero-oligomeric NAD-IDHs from mitochondria. Type II IDHs are mainly comprised of homo-dimeric NADP-IDHs from bacteria and eukaryotes, with a newly found group of homo-dimeric NAD-IDHs from marine bacteria and algae [7,9]. Although IDHs from type I and II subfamilies share similar protein size and topology, their primary sequence identities are less than 15%. The third IDH subfamily members are monomeric, which have longer peptide chains (~740 aa) and lower protein sequence identities (<10%) compared to the other two types of IDHs [7], suggesting that they evolved independently.

Monomeric IDHs have been characterized from various bacteria. It was once recognized that monomeric IDHs are highly specific to NADP^+^, and they cannot use NAD^+^ for catalysis [4,10,11]. We reported a novel type of NAD^+^-specific monomeric IDHs identified from *Campylobacter* species, thus expanding the phylogenesis of the IDH protein family since both NAD^+^ and NADP^+^ specific enzymes exist among all the three IDH subfamilies [7]. Interestingly, further phylogenetic analysis showed that besides the well-characterized monomeric NAD-IDHs and NADP-IDHs clade, a new subgroup of IDHs could be distinguished. IDHs in this clade have a typical monomeric IDH size (~740 aa), and they share about 50% sequence identities with monomeric IDHs. The representative IDH members of this unique subgroup come from some pathogenic bacteria, such as *Acinetobacter* species and *Mycobacterium* species. A previous work demonstrated that the IDH2 (741 aa) from *Mycobacterium tuberculosis* (MtIDH2) forms a stable dimer in solution [12]. This interesting finding, together with the distinguished distribution of these IDHs on the phylogenetic tree, encouraged us to explore the actual structure of this unique subgroup of IDH. In this study, we solved the substrate/coenzyme-free structure of the IDH2 from *Acinetobacter baumannii* (AbIDH2) to 3.0 Å. AbIDH2 is a homodimer, formed through the interactions between the two monomers’ surfaces. To our knowledge, AbIDH2 is the first dimeric structure reported to have originated from the typical monomeric IDH protein family.

## 2. Results and Discussion

### 2.1. Oligomeric State Determination of the Recombinant AbIDH2

**Figure 1:**
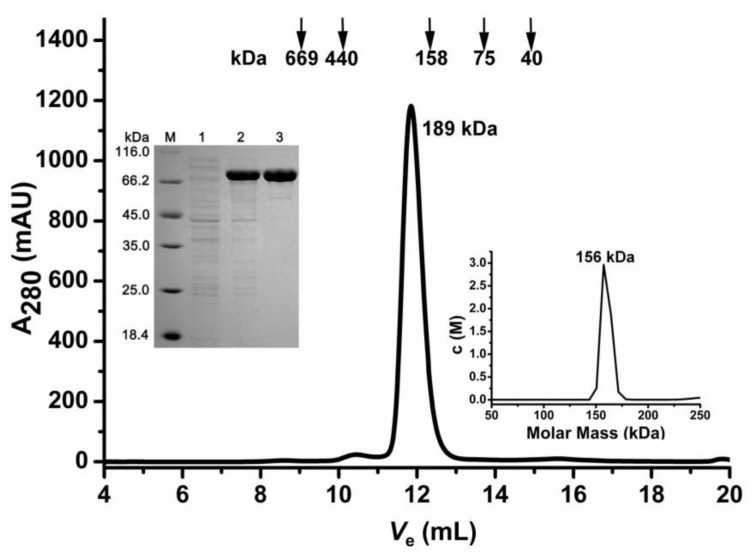
Oligomeric state determination of the recombinant AbIDH2. The flow rate of the size exclusion chromatography was 0.5 mL·min^−1^ and the proteins in the fractions were monitored at 280 nm. *V*_e_ of the recombinant AbIDH2 was 11.78 mL. The upper insert panel shows the protein purity detection by 12% SDS-PAGE. M, protein marker; lane 1, crude extracts of *E. coli* with pET-28b after isopropyl-β-d-thiogalactopyranoside (IPTG) induction; lane 2, crude extracts of *E. coli* with recombinant plasmid pET28-*AbIDH2* after IPTG induction; lane 3, recombinant AbIDH2 after purification. The lower insert panel shows the sedimentation coefficient distribution of the recombinant AbIDH2 at 20 °C.

The recombinant AbIDH2 was acquired through an *E. coli* expression system and then purified through metal affinity chromatography (Figure 1). Purified AbIDH2 was estimated to be around 80 kDa by SDS-PAGE, which compared well with its theoretical calculation (83 kDa). The composition of the polypeptide chain of AbIDH2 suggested a typical monomeric enzyme. However, size exclusion chromatography (SEC) characterization showed that the recombinant AbIDH2 eluted as a single peak and its molecular weight was estimated to be 189 kDa, demonstrating that the AbIDH2 protein exists as homodimer in solution (Figure 1).

SEC is a regular method for estimating protein molecular mass. However, errors can be often introduced by the fact that protein can interact with the column resin in a hydrophobic or electrostatic way [13,14]. In our assay, the inconsistency between the SEC estimation (189 kDa) and the theoretical calculation of the dimeric AbIDH2 (168 kDa) may be caused by the non-ideal interactions between the protein and the SEC media. In order to validate the molecular weight of AbIDH2, we then performed a sedimentation velocity analysis. By applying protein with more than 95% purity, a single analytical centrifugation run was recorded. The distribution of the sedimentation coefficient showed one single and clean peak (Figure 1). The molecular weight of AbIDH2 was calculated to be around 156 kDa. This value is nearly twice the subunit weight, suggesting that the recombinant AbIDH2 exists as a homodimer in solution.

### 2.2. Kinetic Characterization of the Recombinant AbIDH2

Kinetic characterization of the recombinant AbIDH2 demonstrated that its apparent *K*_m_ for NADP^+^ was 94 ± 6 μM or 159 ± 23 μM when using Mn^2+^ or Mg^2+^, respectively (Table 1). No NAD^+^-associated activity was detected, even when using up to 2 mM NAD^+^ as the cofactor, demonstrating that the recombinant AbIDH2 was completely NADP^+^-specific. AbIDH2 needs the divalent cation for catalysis and Mn^2+^ was the most favorable cation, as shown by the fact that the catalytic efficiency for NADP^+^ was 8-fold higher when using Mn^2+^ as compared to that when using Mg^2+^ (Table 1). Although AbIDH2 showed similar apparent *K*_m_ for isocitrate in the presence of Mg^2+^ (21.3 ± 4.2 μM) or Mn^2+^ (20.6 ± 3.1 μM), its catalytic efficiency for isocitrate was also 8-fold higher in the presence of Mn^2+^ compared to achieved that when Mg^2+^ was used as the cofactor (Table 1).

The dimeric AbIDH2, which is composed of two monomeric-IDH-like subunits, shows a much lower affinity towards the coenzyme NADP^+^ (94 μM) when compared to the typical monomeric IDHs, such as *Azotobacter vinelandii* IDH (AvIDH) (5.8 μM), *Corynebacterium glutamicum* IDH (CgIDH) (4 μM), and *Streptomyces lividans* IDH (SlIDH) (2.4 μM) [11,15,16]. Therefore, the catalytic efficiency of the dimeric AbIDH2 was dramatically reduced (0.39 μM^−1^·s^−1^) as compared with those of the well-characterized monomeric IDHs, such as AvIDH (15.9 μM^−1^·s^−1^), CgIDH (22 μM^−1^·s^−1^), and SlIDH (9.6 μM^−1^·s^−1^) [11,15,16]. Furthermore, AbIDH2’s catalytic efficiency is not comparable to those of the typical dimeric NADP-IDHs (with the subunit polypeptide composed of ~410 aa), such as *Escherichia coli* IDH (4.7 μM^−1^·s^−1^), human cytosolic NADP-IDH (2.7 μM^−1^·s^−1^), and yeast cytosolic NADP-IDH (2.5 μM^−1^·s^−1^) [17,18]. Previous works assumed that monomeric IDHs obtained their structure and function through gene duplication and subsequent gene mutation [10,19]. During this process, monomeric IDHs improved the catalytic activity largely to compensate for the loss of a catalytic site caused by monomerization [16,20]. Since the substrate binding pocket and the NADP^+^ associating sites are conserved between AbIDH2 and monomeric IDHs (Figures S1 and S2), the doubling of the catalytic sites in the dimeric AbIDH2 could be expected to improve the enzyme’s activity. However, kinetic analysis of the nonclassical dimeric AbIDH2 demonstrated that the dimerization of the monomeric-IDH-like subunits attenuates the enzyme.

### 2.3. Crystal Structure of the Dimeric AbIDH2

**Figure 2:**
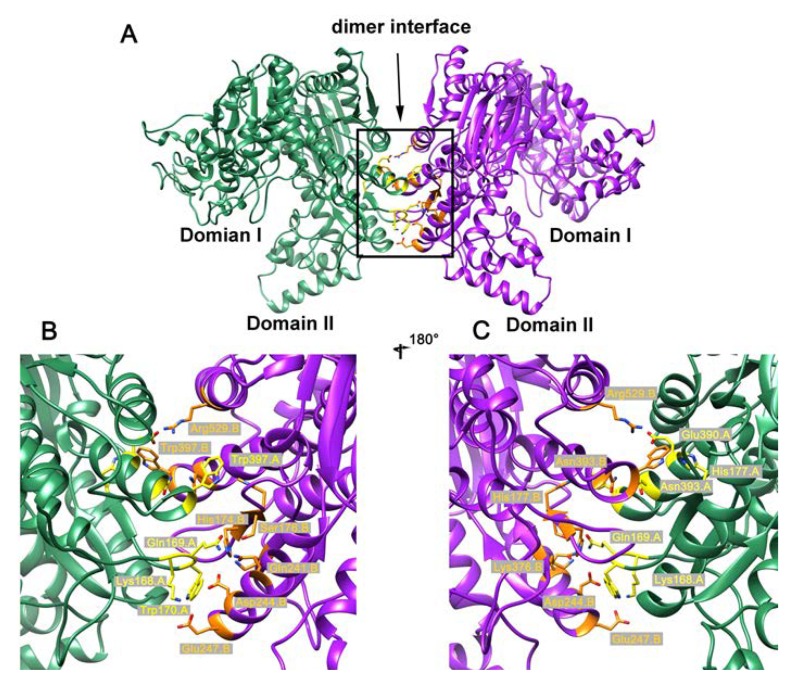
The overall structure of AbIDH2. (**A**) AbIDH2 shows a dimeric structure and each subunit contained a domain I and a domain II. (**B**,**C**) These images show the hydrophobic interactions formed on the interface platform that stabilize the dimeric assembly. The interface platform centers around the three parallel helices (α4, α12, and α17) and one adjacent loop from the domain II in each subunit.

The crystal structure of the substrate/coenzyme-free AbIDH2 was determined at 3.0 Å resolution in space group P4222 with two protomers associated in a dimer per asymmetric unit (Table 2, Figure 2A). Each subunit of AbIDH2 in the refined structure contains 735 amino acid residues (of a total of 745 residues in the AbIDH2 sequence). Electron densities of the first four residues from the N-terminus and the last six residues from the C-terminus were not visible. The AbIDH2 monomer is composed of two distinct domains, as occurs in a typical monomeric IDH structure (Figure 2A) [4,10,19,21]. Domain I constitutes the 305 residues from both the N- and C-termini (amino acids 5–134 and 564–739), and the domain II is formed by the 430 sequential residues (amino acids 135–563). Between the two domains, there is a cleft region that is expected to contain the active site, as occurs in typical monomeric IDHs. In total, 12 α-helices and seven β-strands can be found in domain I, and 18 α-helices and 20 β-strands exist in domain II.

The AbIDH2 protomer and the previously described substrate/coenzyme-free CgIDH share a very similar open conformation structure [4]. The Cα atoms of the two open structures can be superimposed onto each other with a small root-mean-square deviation (RMSD) value of 1.06 Å (Figure S3). The AbIDH2 protomer was then superimposed on the closed structure of AvIDH complexed with a substrate (isocitrate) or coenzyme (NADP^+^), yielding a higher RMSD value of 2.07 and 2.81 Å, respectively (Figure S3). The conformation difference between the open and closed structures was mainly caused by the binding of the substrate or coenzyme at the active site located at the cleft. As the result, domain II is closer to domain I in the AvIDH-isocitrate or AvIDH-NADP^+^ closed structures than that in the AbIDH2 apo structure (Figure S3). These results suggested that this open conformation of AbIDH2 will allow the substrate and coenzyme to enter, following which the two domains will move closer to form an active site for catalysis.

**Figure 3:**
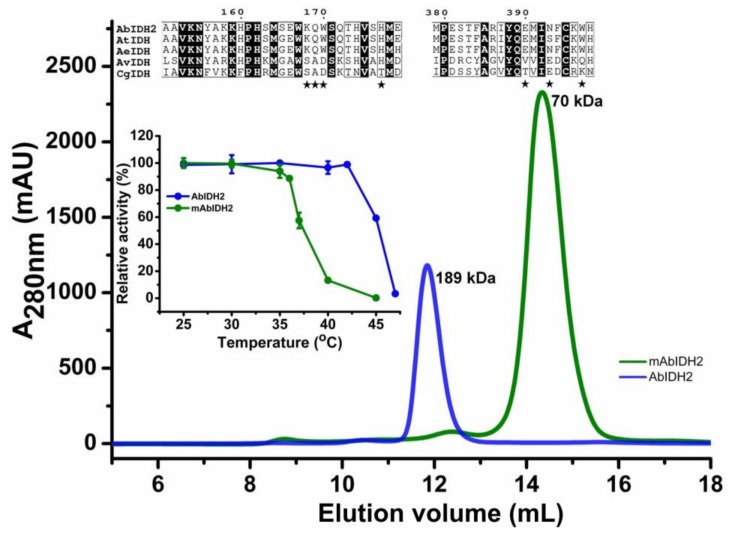
Characterization of the mutant mAbIDH2. The flow rate of the size exclusion chromatography was 0.5 mL·min^−1^ and the proteins in the fractions were monitored at 280 nm. *V*_e_ of the wild-type AbIDH2 and mutant mAbIDH2 were 11.78 and 14.35 mL, respectively. The upper insert panel shows the sequence alignment of the dimeric AbIDH2 with its homologous IDHs from *Azoarcus tolulyticus* (AtIDH, GenBank Accession WP_076602605.1) and *Acidovorax ebreus* (AeIDH, GenBank Accession WP_015913126.1) and two typical monomeric NADP-IDHs from *Azotobacter vinelandii* (AvIDH, GenBank Accession No. BAA11169.1) and *Corynebacterium glutamicum* (CgIDH, GenBank Accession No. WP_011013800.1). The residue numbering in the figure is based on the AbIDH2 sequence. The conserved residues are shaded in black. The residues that are involved in the dimer formation are marked by a pentagram ★. The lower insert panel shows the heat-inactivation profiles of the wild-type AbIDH2 and mutant mAbIDH2 from 25 to 47.5 °C.

Intriguingly, in the AbIDH2 dimer structure, two protomers interact with each other through an interface platform contributed by the domain II of each monomer (Figure 2A). This interface platform is centered around the three parallel helices (α4, α12, and α17) in the domain II and is stabilized by the interactions from the adjacent loops, with a buried surface area of about 3900 Å^2^ (Figure 2A). The dimeric assembly is mainly stabilized by hydrophobic interactions with 16 hydrogen bonds and 11 salt bridges (Figure 2B,C). The Asp390 in α12 interacts with the Arg529 in α17 from the other subunit and forms salt bridges. The Glu247 in α4 interacts with the Lys168 in the loop ahead of α4 from the opposite subunit. Further analysis showed that residues Gln169, Trp170, and His177 from the loop and Asn393 and Trp397 in α12 are directly involved in the formation of the hydrogen bonds that are essential to maintaining the AbIDH2 dimeric structure (Figure 2B,C). Sequence alignment demonstrated that these residues are highly conserved in homologous proteins and the corresponding sites of the typical monomeric IDHs are composed of the other well-conserved set of amino acids (Figure 3).

### 2.4. Mutational Analysis of the AbIDH2

We analyzed the AbIDH2 dimer interface by site-directed mutagenesis. Key residues, Lys168, Gln169, Trp170, His177, Asp390, Asn393 and Trp397, that directly contributed to the dimer formation were simultaneously changed to Ala, generating the mutant enzyme mAbIDH2. Enzymatic characterization showed that mAbIDH2 sustained comparable specific activity (21.9 ± 2 U/mg) to that of the wild-type enzyme (25.4 ± 0.7 U/mg). An analytical gel-filtration experiment demonstrated that the systematic mutations disrupted the AbIDH2 dimeric association, leading to the overwhelming presence of the monomeric mAbIDH2 in solution (Figure 3). These results may rule out the possibility that the dimerization of AbIDH2 serves as a potential method of enzyme regulation, since the monomeric mAbIDH2 is as active as the dimeric AbIDH2.

We further compared the thermostability of the mutant and wild-type enzyme. The monomeric mAbIDH2 showed much worse stability as compared to the wild-type dimeric AbIDH2, demonstrated by the heat-inactivation study in which incubation at 42.5 °C for 20 min caused a dramatic 95% loss of mAbIDH2 activity while no activity loss was observed for AbIDH2 (Figure 3). Furthermore, mAbIDH2 was totally abolished by incubation for 20 min at 45 °C while AbIDH2 still sustained 60% of the activity (Figure 3). The results agree well with previous reports detailing that the monomeric IDHs were not stable above 25 °C [11,22,23]. It is not clear why the IDH2 from *A. baumannii* could bear a much higher temperature than that needed for normal growth. Furthermore, *A. baumannii* could enter a viable nonreplicating state when exposed to the harsh environments where nutrients were low [24,25]. The bacteria can be resuscitated upon infecting the host cell and continue growth and pathogenicity. It is not known whether *A. baumannii* proteins are thermostable in general or if it is a special trait of some few, like AbIDH2. In the latter case, it will be necessary to investigate whether AbIDH2 would contribute to the survival of the bacteria in unfavorable environments.

### 2.5. Redefining the Phylogenesis of the Monomeric IDH Subfamily

**Figure 4:**
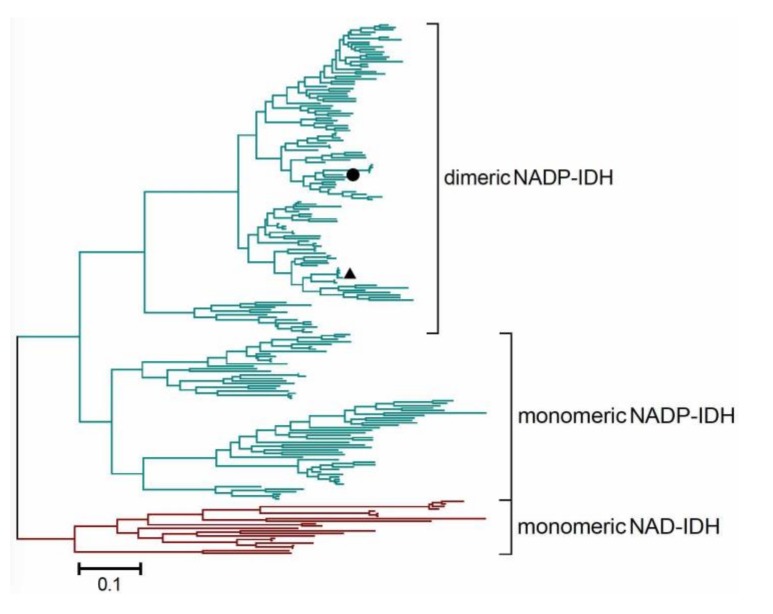
Molecular phylogenetic analysis of the type III IDH protein subfamily by the Maximum Likelihood method. The tree is drawn to scale, with branch lengths measured in the number of substitutions per site. The analysis involved 216 IDH sequences. All positions containing gaps and missing data were eliminated. Evolutionary analyses were conducted in MEGA7. The dimeric AbIDH2 and MtIDH2 (the second NADP-IDH from *M*. *tuberculosis*) were marked by closed circle (●) and triangle (▲), respectively.

We previously divided the IDH protein family into three subfamilies: type I IDHs, type II IDHs, and monomeric IDHs [7]. Two kinds of monomeric IDHs, NADP-IDH and NAD-IDH, can be distinguished in the monomeric IDH subfamily according to their coenzyme specificity. In this study, we redefined the formal monomeric IDH subfamily by reporting a new subtype of dimeric IDHs which were composed of two monomeric-IDH-like subunits. This novel group of dimeric IDHs was represented by the second NADP-IDH from the pathogen *A*. *baumannii* (AbIDH2) (Figure 4). The crystal structure of the substrate/coenzyme-free AbIDH2 was determined and its dimeric state was explored. Taking into consideration the fact that both monomeric and dimeric IDHs were found in this subfamily, it is inappropriate to name this subfamily as “monomeric IDHs” as before. Instead, we redefined this IDH subfamily as “type III IDHs”.

Numerous IDHs with a size of around 85 kDa were grouped together with AbIDH2 during the rebuilding the phylogenetic tree of the type III IDH subfamily (Figure 4). As AbIDH2 was demonstrated to be a homodimeric protein by our crystallography study, this new clade was therefore defined as the type III “dimeric NADP-IDH”. We, for the first time, expanded the original “monomeric IDH” protein subfamily by adding the new subgroup of dimeric IDHs. Interestingly, the second NADP-IDH from another pathogen, *Mycobacterium tuberculosis* (MtIDH2), has also been biochemically identified as a homodimer composed of two subunits with a size of 86 kDa [12]. Consistently, our evolutionary analysis clearly showed that MtIDH2 grouped with the type III dimeric IDHs, not with the monomeric IDHs (Figure 4).

Our study suggested that both type III monomeric IDHs and dimeric IDHs are widely distributed in bacteria. Monomeric IDHs have been demonstrated to be the most efficient IDHs among the ancient IDH protein family [10,11]. The newly defined type III dimeric IDHs, on the contrary, are latent in catalyzing. It will be interesting to look into the question of why dimerization attenuates the robust monomeric IDH. Although the apo structure of AbIDH2 reveals how the dimerization occurred between two monomeric IDH-like subunits, further efforts are required, such as the structure of the AbIDH2 complexed with the substrate and coenzyme, to elucidate the catalytic mechanism of this novel type III dimeric IDH.

## 3. Materials and Methods

### 3.1. Cloning, Expression, and Purification of AbIDH2

A full-length *AbIDH2* gene from *A*. *baumannii* was cloned into pET28b (Invitrogen, Carlsbad, CA, USA). Plasmid was then transformed into *E. coli* BL21 (DE3) and the recombinant protein was overexpressed. The transformed cells were grown on Luria-Bertani (LB) agar plate containing 100 μg mL^−1^ kanamycin. A single colony was picked and grown in LB medium containing 100 μg mL^−1^ kanamycin until OD_600_ reached 0.6 at 37 °C. After induction by 0.5 mM isopropyl-β-d-thiogalactopyranoside (IPTG), the cells were further grown for 20 h at 22 °C and harvested by centrifugation. *E. coli* BL21 cells were resuspended in lysis buffer containing 50 mM NaH_2_PO_4_ pH 7.5, 300 mM NaCl, and then disrupted by sonication. The recombinant AbIDH2 with 6× His-tag on its N-terminus was purified using BD TALON Metal Affinity Resin (Clontech, LaJolla, CA, USA) according to the manufacturer’s instructions. Protein was further purified by gel filtration on a Hi-Load 16/60 Superdex 200 column (Amersham Biosciences, Uppsala, Sweden). The major peak fractions were collected and concentrated for crystallization. Mutations were introduced into AbIDH2 by overlap extension, PCR-based, site-directed mutagenesis. Mutant AbIDH2 was expressed and purified as the wild-type AbIDH2.

### 3.2. SEC Chromatography

Analytical gel-filtration experiments were performed with a HiLoad™ 10/300 Superdex 200 column (Amersham Biosciences). A 1.5-mL aliquot of purified proteins were eluted using 50 mM NaH_2_PO_4_ pH 7.5, 300 mM NaCl. Protein molecular mass was evaluated from a calibration curve using ovalbumin (45 kDa), conalbumin (75 kDa), aldolase (158 kDa), ferritin (440 kDa), and thyroglobulin (669 kDa).

### 3.3. Sedimentation Velocity

Analytical ultracentrifugation (AUC) analysis was conducted with a Beckman XL-A analytical ultracentrifuge, using a four-hole An-60 Ti rotor and a test time interval of 3 min. The reference loading volume was 410 μL, and the sample loading volume was 400 μL. In a typical experiment, 280 absorbance profiles were recorded at 20 °C and 45,000 *g*. The test mode was based on the sedimentation velocity, and a continuous c(s) distribution was applied in the analysis mode. The derivative profiles were used to calculate the experimental sedimentation coefficient (*s*_exp_). The data were also analyzed using the Svedberg program [26]. The Sednterp program (http://bbri.org/RASMB/rasmb.html) was used to calculate the partial specific volume (*v*_2_), solvent density (ρ), and viscosity (η). The corrected coefficient, *s*_20,w_, was calculated using the following equation: *s*_20,w_ = *s*_exp_ (η/η_w,20_) (1 − ρ_w,20_·× *v*_2_)/(1 − ρ*·*× *v*_2_).

### 3.4. Enzyme Assay and Kinetic Determination

IDH activity assays were carried out at 25 °C in buffer containing 35 mM Tris-HCl (pH 7.5), 2 mM MgCl_2_ or MnCl_2_, 1.5 mM DL-isocitrate, and 1.0 mM NADP^+^. An NADPH increase was detected at 340 nm with a thermostated Cary 300 UV-Vis spectrophotometer (Varian, Santa Clara, CA, USA), using a molar extinction coefficient of 6.22 mM^−1^·cm^−1^. One unit of enzyme activity was defined as the reduction of 1 µM of NADP^+^ per minute. Protein concentrations were determined using the Bio-Rad protein assay kit (Bio-Rad, Hercules, CA, USA) with bovine serum albumin as the standard. The Michaelis constant (*K*_m_) values of the wild-type and mutant enzymes for NAD^+^ and NADP^+^ were measured by fixing the isocitrate concentration at 1.0 mM with varying cofactor concentrations. Apparent maximum velocity (*V*_max_) and *K*_m_ values were calculated by nonlinear regression using Prism 5.0 (Prism, San Diego, CA, USA). All kinetic parameters were obtained from at least three measurements.

### 3.5. Crystallization and Structure Determination

The recombinant AbIDH2 was purified and then concentrated to 10 mg/mL for crystal growing. Protein crystals were acquired by the vapor diffusion using the mother liquid consisting of 0.1 M HEPES pH 7.5, 14% 2-propanol, 20% glycerol, 10% PEG4000, and 40 mM MgCl_2_, and then were flash-frozen at 100 K in a nitrogen gas stream in the cryoprotectant consisting of the mother solution supplemented with 30% ethylene glycol. The crystals diffracted to 3.0 Å resolution and the data were collected at beam line 17U1 and 19U1 of the Shanghai Synchrotron Radiation Facility. The atomic coordinates of the *C. glutamicum* IDH (CgIDH) (PDB ID 2B0T) were used as the search model for the molecular replacement method by program PHASER [27]. The model was manually built through the utilization of COOT [28]. The structure refinement was carried out through the use of Phenix [29]. The coordinates and structure factors of AbIDH2 were deposited to Protein Data Bank with an accession number of 5Z16.

### 3.6. Sequence Alignments and Phylogenetic Analysis

The structure-based amino acid sequence alignment was performed by using the Clustal X program and ESPript 3.0 web tool [30,31]. The AbIDH2 protein sequence was used as bait to find homologous sequences by performing a BLAST Link search. A bunch of similar IDH sequences were chosen for phylogenetic analysis. The bootstrapped neighbor-joining tree was constructed with the MEGA 7 software, based on the sequence alignment by Clustal X program [30,32].

## Figures and Tables

**Table 1:** The kinetic parameters of the recombinant AbIDH2.

Enzyme	Isocitrate			NADP^+^		
	*K*_m_ (μM)	*k*_cat_ (s^−1^)	*k*_cat_/*K*_m_ (μM^−1^·s^−1^)	*K*_m_ (μM)	*k*_cat_ (s^−1^)	*k*_cat_/*K*_m_ (μM^−1^ s^−1^)
AbIDH2 (Mg^2+^)	21 ± 4	5.2 ± 0.3	0.24	159 ± 23	7.4 ± 0.6	0.05
AbIDH2 (Mn^2+^)^5^	21 ± 3	39.2 ± 2.1	1.9	94 ± 6	36.9 ± 1.2	0.39

**Table 2:** Data collection and refinement statistics for AbIDH2.

Statistics		AbIDH2
Data Collection	Space group	P4 222
	Cell dimensions	
	*a*, *b*, *c* (Å)	137.16, 137.16, 238.13
	α, β, γ (°)	90.00, 90.00, 90.00
	Wavelength (Å)	0.97776
	Resolution (Å) ^a^	48.54–3.0 (3.05–3.0)
	*R*_sym_ or *R*_merge_	0.132 (0.402)
	*Average I/*σ(*I*)	3.2 (1.96)
	Completeness (%)	99.96 (100)
	Redundancy	3.3 (3.2)
Refinement	Resolution (Å)	3.0
	No. reflections	46,278 (4541)
	*R*_work_/*R*_free_	0.205 (0.245)/0.276 (0.323)
	No. atoms	
	Protein	11,408
	Ligand/ion	12
	Water	369
	*B*-factors	
	Protein	54.2
	Ligand/ion	48.7
	Water	57.2
	R.m.s deviations ^b^	
	Bond lengths (Å)	0.0114
	Bond angles (°)	1.688

^a^ Highest-resolution shell is shown in parentheses; ^b^ R. m. s deviation, root-mean-square deviation.

## Supplementary Materials

Supplementary materials can be found at http://www.mdpi.com/1422-0067/19/4/1131/s1.
