# Supplementary material for: Crystal Structure of the Isocitrate Dehydrogenase 2 from Acinetobacter baumannii (AbIDH2) Reveals a Novel Dimeric Structure with Two Monomeric-IDH-Like Subunits
Source: Int J Mol Sci. 2018 Apr 10;19(4):1131. doi: 10.3390/ijms19041131 (PMC5979607; doi:10.3390/ijms19041131)
Supplement: Supplementary file 1 [file ijms-19-01131-s001.pdf]

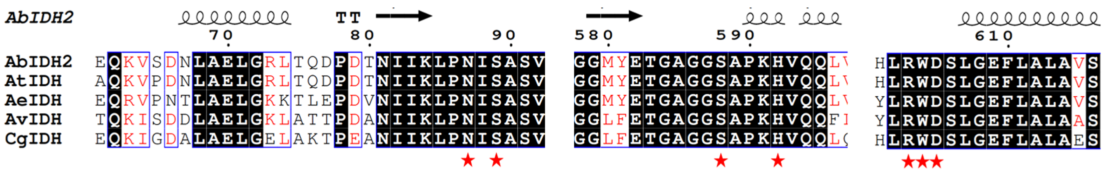

Fig. S1

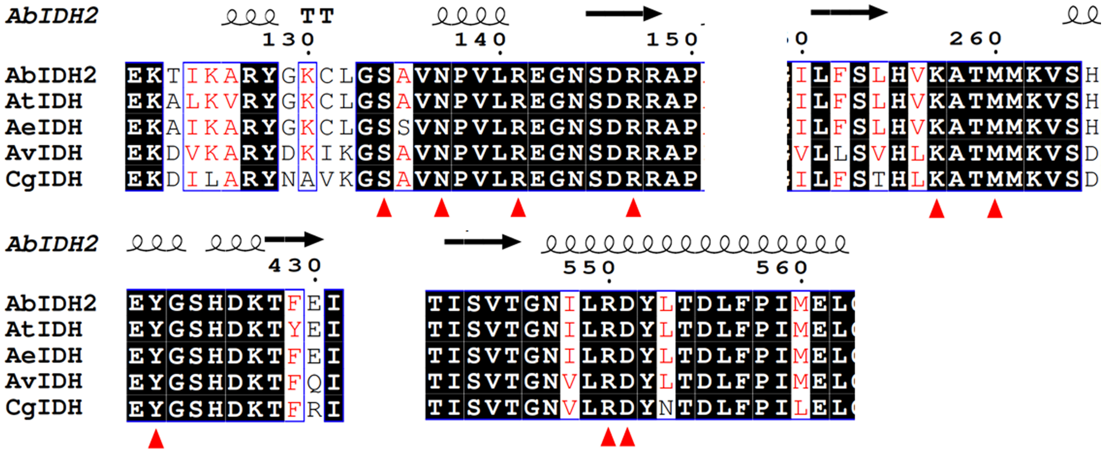

Fig. S2

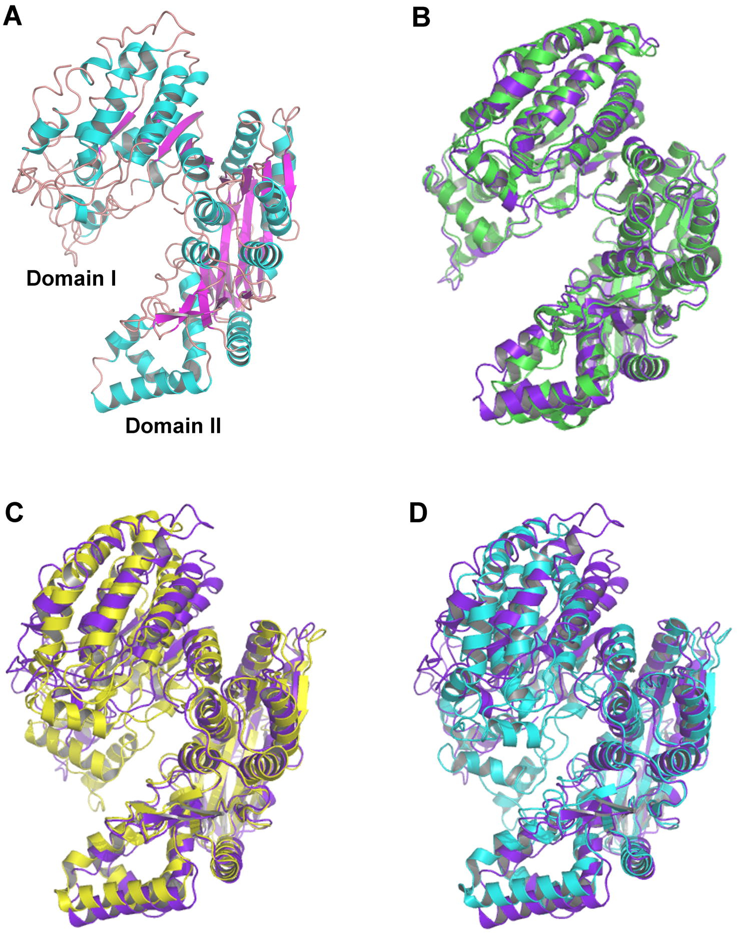

Fig. S3

Figure legends

Fig. S1. The sequence alignment of the dimeric AbIDH2 with its homologous IDHs from *Azoarcus tolulyticus* (AtIDH, GenBank Accession WP_076602605.1) and *Acidovorax ebreus* (AeIDH, GenBank Accession WP_015913126.1) and two typical monomeric NADP-IDHs from *Azotobacter vinelandii* (AvIDH, GenBank Accession No. BAA11169.1) and *Corynebacterium glutamicum* (CgIDH, GenBank Accession No. WP_011013800.1). The residue numbering in the figure is based on AbIDH2 sequence. The conserved residues are shaded by black. The residues that involved in $NADP^+$ binding are marked by red pentagram★.

Fig. S2. The sequence alignment of the dimeric AbIDH2 with its homologous IDHs from *Azoarcus tolulyticus* (AtIDH, GenBank Accession WP_076602605.1) and *Acidovorax ebreus* (AeIDH, GenBank Accession WP_015913126.1) and two typical monomeric NADP-IDHs from *Azotobacter vinelandii* (AvIDH, GenBank Accession No. BAA11169.1) and *Corynebacterium glutamicum* (CgIDH, GenBank Accession No. WP_011013800.1). The residue numbering in the figure is based on AbIDH2 sequence. The conserved residues are shaded by black. The residues that involved in substrate binding are marked by red triangle▲.

Fig. S3. (A), Overall AbIDH2 monomer structure is resolved into domain I and domain II. There are 12 α-helices and 7 β-strands in domain I, and 18 α-helices and 20 β-strands in domain II. (B), Superimposition of apo-AtIDH2 (purple) and apo-CgIDH (green, 2b0t) secondary structures. (C), Superimposition of apo-AtIDH2 (purple) and AvIDH-isocitrate (yellow, 1jtw) secondary structures. (D), Superimposition of apo-AtIDH2 (purple) and AvIDH-$NADP^+$ (cyan, 1j1w) secondary structures.
